# Concurrent Composite Lymphomas Collectively Bearing Three Diagnostic Entities of Shared Clonal Origin

**DOI:** 10.1097/HS9.0000000000000705

**Published:** 2022-03-29

**Authors:** Waleed Alduaij, Laura K. Hilton, Muntadhar Al Moosawi, Susana Ben-Neriah, Barbara Meissner, Merrill Boyle, Kelly Mekwunye, David W. Scott, Heather A. Leitch, Jeffrey W. Craig

**Affiliations:** 1 Centre for Lymphoid Cancer, British Columbia Cancer, Vancouver, British Columbia, Canada; 2 Clinician Investigator Program, Department of Medicine, University of British Columbia, Vancouver, British Columbia, Canada; 3 Hematological Pathology, Department of Pathology and Laboratory Medicine, University of British Columbia, Vancouver, British Columbia, Canada; 4 Division of Medical Oncology, University of British Columbia, Vancouver, British Columbia, Canada; 5 Hematology, St. Paul’s Hospital, Vancouver, British Columbia, Canada; 6 Division of Hematology, Department of Medicine, University of British Columbia, Vancouver, British Columbia, Canada; 7 Department of Pathology and Laboratory Medicine, British Columbia Cancer, Vancouver, British Columbia, Canada

Composite lymphoma (CL) is the coexistence of two or more distinct lymphoma subtypes in a single biopsy, whereas discordant lymphoma (DL) represents their occurrence at different anatomical sites. CL and DL were historically considered rare, but a recent study showed that 12.9% of all newly diagnosed diffuse large B-cell lymphomas (DLBCL) are CL/DL with a concurrent indolent lymphoma component.^1^ CL/DL may comprise two different lymphoma entities that share a clonally related ancestor or represent 2 independent, clonally unrelated tumors.^2^ Classically, CL was shown to consist of Hodgkin lymphoma (HL) and B-cell non-HL with a common germinal center B-cell (GCB) precursor,^3^ though various other combinations of lymphomas have since been described.^2^

There are no clear guidelines on the management of CL/DL due to the diversity of possible CL/DL components and the exclusion of such patients from most clinical trials. Meaningful staging of CL/DL can also be challenging, and prognostication compared to their respective lymphoma components in isolation is often unclear. CL/DL with DLBCL and an indolent component were previously thought to have a worse prognosis than DLBCL alone, as the DLBCL component was presumed to arise through the transformation of the indolent component. However, clinical outcome studies have failed to demonstrate differences in overall survival.^1,4^ Nevertheless, a higher risk of relapse was found in CL/DL due to relapse of the indolent component.^4^

Here, we report a unique case featuring three different GCB-derived lymphoma subtypes presenting simultaneously as a pair of composite tumors in separate anatomical locations. B-cell clonality assays identified the same clonal immunoglobulin kappa light chain (*IGK*) rearrangement in all tumor components, consistent with a shared clonal origin. Targeted capture-based sequencing further characterized this clonal relationship, supporting the development of anatomically and morphologically distinct tumor components through divergent clonal evolution from a common ancestral cell.

A previously healthy, human immunodeficiency virus–negative, 48-year-old man presented with progressive dysphagia and stridor. Flexible laryngoscopy revealed a large mass at the base of the tongue, with impending airway obstruction. Tracheostomy was performed with a concomitant biopsy of the tongue mass. An excisional biopsy of an enlarged cervical lymph node was also performed to increase diagnostic yield.

The cervical lymph node biopsy (Figure 1A) revealed distinct areas of classic HL (cHL) and DLBCL. The cHL component contained scattered Hodgkin and Reed-Sternberg (HRS) cells, including frequent lacunar forms, in a mixed inflammatory background featuring numerous small lymphocytes, eosinophils, histiocytes, and plasma cells (Figure 1B, inset: high-power view of a representative HRS cell). By immunohistochemistry (IHC), the HRS cells were strongly positive for CD30 (Figure 1C), weakly positive for PAX5 and included rare cells positive for CD15 (not shown). In situ hybridization for Epstein-Barr virus RNA (EBER-ISH) was positive in the HRS cells. CD45, CD19, CD20, and CD79a stains were negative. The DLBCL component consisted of a sheet-like proliferation of large, mononuclear lymphoid cells (Figure 1D) that stained strongly for CD45, PAX5, CD19, CD20 (Figure 1E), and CD79a and exhibited ≥30% reactivity for CD10, BCL6, and MUM1, consistent with a GCB phenotype by Hans algorithm.^5^ Additional IHC showed that the DLBCL component was positive for MYC but negative for BCL2, CD30, and CD15. EBER-ISH was also negative in this cell population.

**Figure 1.**
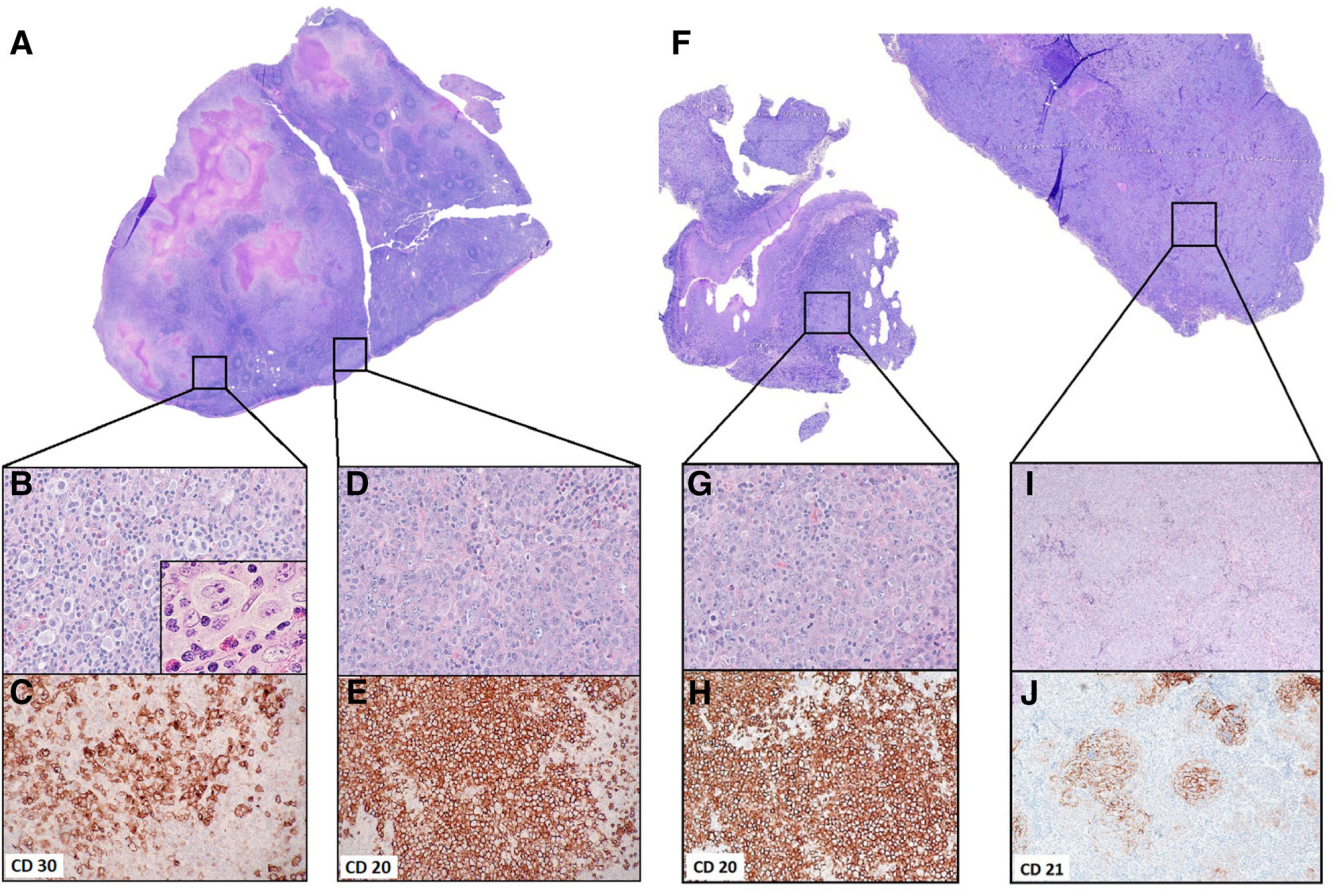
**Morphological features of the composite lymphomas identified in lymph node and tongue biopsies** (A) H&E–stained whole slide image of the cervical lymph node biopsy (maximum tissue dimension = 1.8 cm). (B) Lymph node area showing cHL, OM ×40, H&E, inset: high-power view of a representative HRS cell OM ×100 oil immersion. (C) cHL, OM ×20, CD30 highlighting HRS cells. (D) Lymph node area showing DLBCL, OM ×40, H&E. (E) DLBCL, OM ×20, CD20. (F) H&E-stained whole slide image of the tongue mass biopsy (image width = 1.1 cm). (G) Tongue mass area showing DLBCL, OM ×40, H&E. (H) DLBCL, OM ×10, CD20. (I) Tongue mass area showing FOLL3B, OM ×10, H&E. (J) FOLL3B, OM ×10, CD21 highlighting follicular dendritic cell meshwork. cHL = classic Hodgkin lymphoma; DLBCL = diffuse large B-cell lymphomas; FOLL3B = follicular lymphoma grade 3B; H&E = hematoxylin and eosin; HRS = Hodgkin Reed-Sternberg; OM = objective magnification.

The tongue biopsy consisted of discrete areas of DLBCL and follicular lymphoma (FL), grade 3B (FOLL3B, Figure 1F). The DLBCL component had similar morphologic and immunophenotypic features to the cervical lymph node DLBCL (Figure 1G, H). The FOLL3B component also comprised a uniform population of atypical centroblastic cells, but unlike the DLBCL component, it exhibited a nodular growth pattern consistent with neoplastic follicle formation (Figure 1I). The presence of intact CD21-positive follicular dendritic cell meshwork confirmed the histologic impression (Figure 1J). Benign residual GCBs were not detected, and the biopsy did not contain remnants of lingual tonsil. EBER-ISH was negative throughout this specimen. Suppl. Table S1 provides comprehensive IHC data using the same panel of antibodies applied to all four tumor components.

To investigate the clonal relationships among the lymphoma subtypes, genomic DNA was extracted from 1-mm core fragments obtained from regions of the paraffin-embedded tissue blocks corresponding to each separate tumor component as highlighted by the squares in Figure 1A, F. Multiplex polymerase chain reaction (PCR) analysis using *IGH* VH-JH BIOMED-2 primer sets^6^ yielded normal peak distributions reflecting only the polyclonal B-cell background populations present within each of the 4 independent samples (data not shown). Therefore, we extended our analysis to include the *IGK* locus, relying again on the BIOMED-2 primer design. Although the Vk-Jk reaction (tube A) detected no clonal peaks (data not shown), the Vk-Kde/intron-Kde reaction (tube B) identified a single prominent peak at 377 base pair (bp) length amplified by the Vk2f/Vk4/Vk5-Kde primer set,^6^ consistent with a clonal B-cell population (Vk-Kde 3, Figure 2A, B). The same peak was identified in all 4 tumor components, including the nodal cHL, albeit at a lower amplitude in keeping with the lower tumor cell content of cHL (Figure 2A, top panel). The presence of the same *IGK* clonal peak in all 4 tumor components strongly suggested a shared clonal B-cell origin among all 3 histologic lymphoma subtypes.

**Figure 2.**
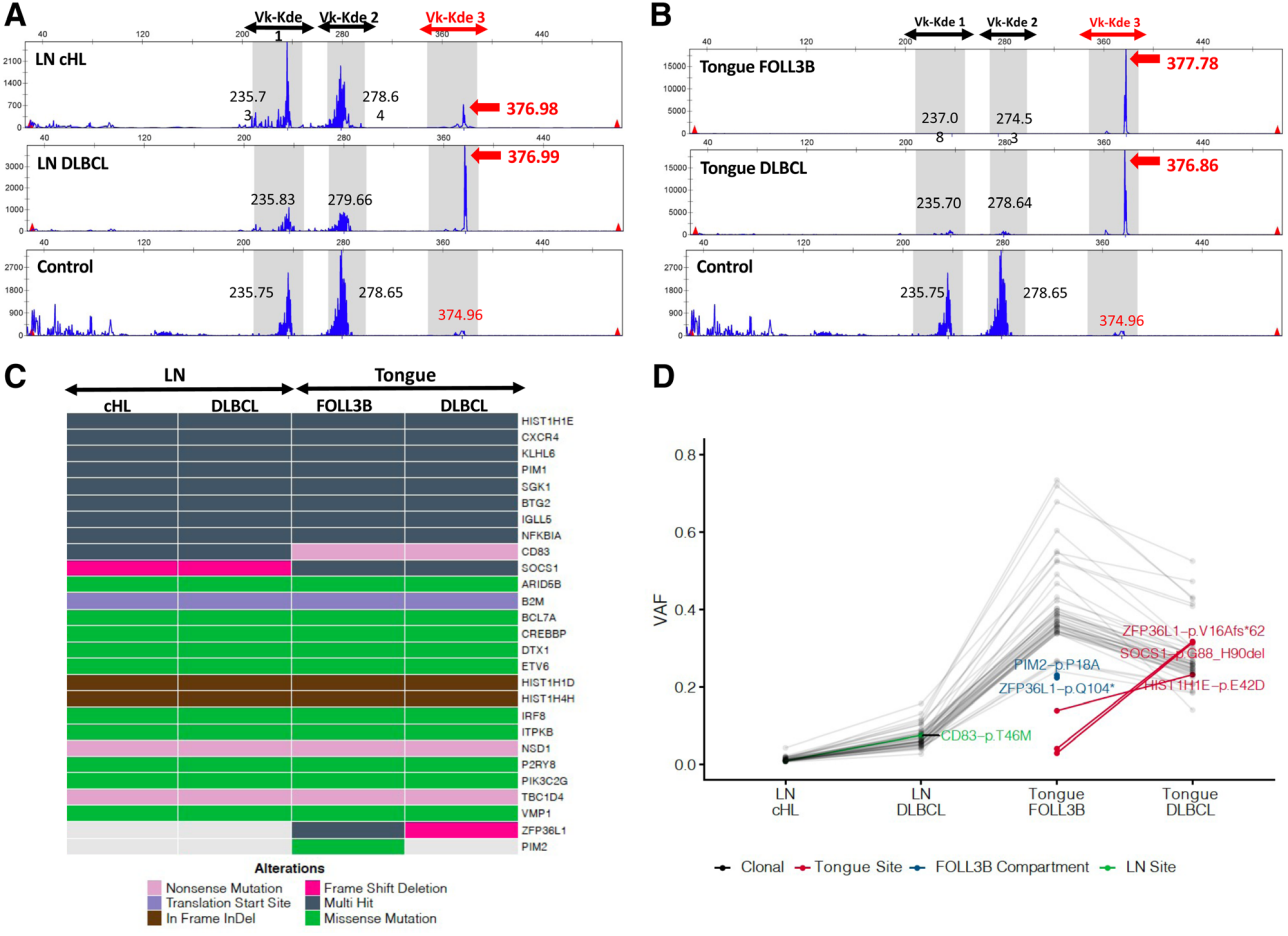
**Clonal relationship among the 4 tumor components.** (A and B) *IGK*-rearrangement–based B-cell clonality analysis of composite tumor components using *IGK* Vk-Kde/intron-Kde BIOMED-2 primer sets in (A) the cervical LN cHL and DLBCL components, and (B) the base of tongue FOLL3B and DLBCL components. Genomic DNA was extracted from 1.0 mm core fragments obtained from regions of the paraffin-embedded tissue blocks corresponding to each highlighted tumor component. Multiplex PCR analysis was performed using *IGK* BIOMED-2 primer sets (IdentiClone *IGK* Gene Clonality Assay; Invovoscribe; Catalog # 91020021). PCR products were resolved by capillary electrophoresis and analyzed using associated software. Polyclonal control DNA (Invovoscribe, Catalog # 40920010) was used as a negative control shown in the lower panel. The *IGK* Vk-Kde/intron-Kde reaction yields 3 discrete collections of PCR products corresponding to the following primer sets and their anticipated PCR product size ranges: Vk-Kde 1: Vk1f/6/Vk7-Kde, 210-250 bp, Vk-Kde 2: Vk3/intron-Kde, 270-300 bp, and Vk-Kde 3 (in red): Vk2f/Vk4/Vk5-Kde, 350-390 bp. Red arrows highlight the 377 bp clonal peak identified in all tumor components in the Vk-Kde 3 window, consistent with a shared B-cell clonal origin. Black numbers provide the lengths (bp) of maximal amplitude peaks within the non-highlighted portions of the *IGK* Vk-Kde/intron-Kde reaction. (C and D) Targeted capture sequencing performed on the extracted genomic DNA using a customized panel of 312 lymphoma-related genes. (C) Oncoplot characterizing the type of genetic alterations detected in each tumor component. “Multi-hit” indicates more than one alteration in the same gene and are all shared except for *HIST1H1E*-pE42D, which is unique to the tongue biopsy. Refer to Suppl. Table S3 for individual mutations. (D) VAF of individual mutations. VAF reflects the tumor content of each sample, which is predictably lowest for the cHL component. Connected gray points indicate clonal mutations shared across all tumor components. Connected green and red points highlight mutations exclusive to the lymph node and tongue biopsies, respectively. Blue points highlight mutations unique to the FOLL3B component. cHL = classic Hodgkin lymphoma; DLBCL = diffuse large B-cell lymphoma; FOLL3B = follicular lymphoma grade 3B; LN = lymph node; PCR = polymerase chain reaction; VAF = variant allele frequency.

To allow further characterization of the individual tumor components beyond the capabilities of routine clinical workflow, the patient provided written informed consent for additional analysis to be performed in a research setting. Fluorescence in situ hybridization (FISH) analysis using break-apart probes showed no evidence of *MYC* rearrangement. In contrast, a *BCL2* rearrangement was identified with a consistent pattern across all tumor components (2 fused signals, one 5′*BCL2* and one 3′*BCL2* probe signals), including the HRS cells in the nodal cHL component (Suppl. Figure S1), strengthening the argument for a shared clonal origin.

In order to more precisely characterize the clonal relationships among the tumor components, we next performed targeted deep sequencing of the extracted genomic DNA using a customized gene panel derived from previously described hybridization capture panels for DLBCL^7^ and Hodgkin/gray zone lymphoma.^8^ The resultant panel of 312 genes (Suppl. Table S2) also covers the majority of genes implicated in FL.^9^ Each sample was sequenced to a minimum depth of 1400× (3200× in the cHL component). Fifty-four mutations were identified in 27 genes, with more than 1 mutation detected in 13 genes. The majority (48 mutations in 23 genes) were detected within all 4 tumor components, further supporting their shared clonal origin (Figure 2C, Suppl. Table S3). The variant allele frequency reflects the tumor content of each sample, which is predictably lowest for the cHL component. Four additional mutations were unique to each anatomic site: *CD83*-p.T46M in the cervical lymph node and *ZFP36L1*-p.V16Afs*62, *SOCS1*-p.G88_H90del and *HIST1H1E*-p.E42D in the tongue, in keeping with clonal divergence following the anatomical dissemination of a shared clonal precursor. The remaining 2 mutations (*PIM2*-p.P18A and *ZFP36L1*-p.Q104) were exclusive to the FOLL3B sample (Figure 2D), providing a genetic correlate to this architecturally distinct tumor component.

Computed tomography scanning confirmed stage IV disease, including an 11.3 cm mass in the cecum. A bone marrow biopsy showed no evidence of lymphoma. Because DLBCL was present in both diagnostic specimens and warranted immediate action to avoid airway compromise, the patient was treated with 6 cycles of rituximab plus cyclophosphamide, doxorubicin, vincristine, and prednisone (R-CHOP). Treatment course was complicated by terminal ileum obstruction requiring right hemicolectomy, and analysis of the surgical specimen showed evidence of DLBCL in a small focus of viable tumor. He ultimately achieved a complete remission, sustained at 24 months from diagnosis.

Although CL/DL with 2 distinct lymphomas is a well-described phenomenon, the diagnosis of 3 lymphomas simultaneously is a much rarer occurrence, with only a few cases reported in the literature. Steinhoff et al^10^ described the occurrence of 3 lymphomas in 1 patient with peripheral T-cell lymphoma manifesting as a composite tumor with both marginal zone B-cell lymphoma in the skin, and cHL in a cervical lymph node. Molecular analyses showed these to be unrelated processes. Nishioka et al^11^ reported a patient with DLBCL in the esophagus and a composite of cHL and FL in an inguinal node. The latter 2 entities were derived from a common GCB precursor. Four cases of triple CL in a single lymph node have been reported, with each showing variable histologies.^12–15^ Only one of these successfully demonstrated a clonal relationship among its components.^12^

A fundamental question in CL/DL is whether the tumor components are clonally related, arising from a common ancestor, or instead, are de novo independent events. *IGH* PCR–based B-cell clonality assays are often used in a clinical setting to address this question, but the results are not always definitive. In our case, no clonal *IGH* rearrangement was identified in any of the independently tested tumor components, highlighting the inherent limitations of these assays. Sensitivity rates as low as 84% in FL and 79% in DLBCL have been reported.^16^

The primary mechanism underlying false-negative immunoglobulin gene (Ig-gene) clonality results is somatic hypermutation (SHM) of the immunoglobulin variable regions, leading to ineffective primer binding and failure of PCR amplification.^17^ GCB malignancies, such as the lymphomas identified in this case, have undergone SHM and are therefore susceptible to these changes. Currently, the best available strategy to increase diagnostic sensitivity is consensus primers that target multiple Ig-gene segments. Given that this approach will be limited by the quantity of diagnostic material available and cost in routine clinical practice, sequential or reflexive analysis using *IGH* VH-JH and *IGK* primer sets may be employed. This focused strategy can establish clonality in 98% of histologically confirmed mature non-Hodgkin B-cell malignancies,^16^ as demonstrated here.

However, the sensitivity of Ig-gene PCR remains problematically low in cHL due to the scarcity of neoplastic cells and the presence of a dense polyclonal lymphoid background, resulting in sensitivity rates of roughly 25% when using paired *IGH* VH-JH and *IGK* analyses.^18^ Without HRS cell microdissection, it is difficult to completely exclude the possibility of DLBCL cross-contamination as an explanation for our *IGK* and targeted deep sequencing findings in the nodal cHL sample. However, identification of the shared *BCL2* rearrangement in diagnostic HRS cells by FISH argues strongly against this possibility.

Targeted deep sequencing tailored to GCB malignancies provided deeper insights into the molecular underpinnings of this patient’s disease. Similar testing has not been described previously in patients with 3 simultaneous lymphoma diagnoses to the best of our knowledge. Of the detected mutations, 88% were found in all tumor components, reinforcing their shared clonal origin. Among these were shared mutations in *SOCS1*, *SGK1*, and *NFKBIA*, which contribute to Janus kinase-signal transducer and activator of transcription proteins,^19^ phosphoinositide 3-kinase,^20^ and nuclear factor kappa-lightchain-enhancer of activated B cells signaling, respectively. Mutations within these and several other genes identified in all 4 tumor components (eg, *ITPKB*, *CD83*, *P2RY8*, *HIST1H1D*, *HIST1H1E*, *BCL7A*, *DTX1*) are particularly enriched in genetically-defined subsets of predominantly GCB-type DLBCL tumors, variably referred to as ST2,^21^ C4,^22^ and SOCS1/SGK1,^23^ associated with a favorable outcome.

We found evidence of clonal divergence with respect to anatomic location (ie, mutations unique to the neck and tongue biopsy sites, respectively), including mutational differences between the histologically similar nodal and tongue DLBCL components. Spatial genetic heterogeneity is a well-established phenomenon in solid cancers and has been described in FL.^24,25^ Furthermore, the identification of mutations unique to the FOLL3B component supports the notion that despite the cytomorphologic overlap between the FOLL3B and DLBCL in this case, these components are indeed genetically divergent and do not simply represent different architectural manifestations of the same process. The precise molecular mechanisms governing the transition between FOLL3B and DLBCL have not been defined, so it remains unclear how mutations in *PIM2* and *ZFP36L1* would directly contribute to this phenomenon.

In contrast, despite the marked morphological difference between the nodal DLBCL and cHL components, no mutational differences were identified within the 312 genes examined. This may reflect the low tumor cell content of cHL, which limits the ability to detect all somatic variants despite achieving high sequencing depth. Genetic aberrations beyond the genomic coverage of targeted capture such as copy number, structural variants, intronic mutations and exonic mutations outside the targeted hot-spots were not evaluable with our methods but provide ample room for unexplored genetic divergence. The exclusive EBER positivity of the nodal cHL component suggests that extrinsic viral DNA elements are likely to be important in this regard.

Epstein-Barr virus (EBV) helps rescue crippled GCBs from apoptosis and contributes significantly to early lymphomagenesis in EBV+ cHL.^26^ The viral oncoprotein, latent membrane protein-1, induces altered transcriptional patterns characteristic of cHL^27^ and drives the morphologic changes characteristic of Reed-Sternberg cells.^28,29^ Because EBV can be progressively lost from lymphoma clones after mediating initial cellular transformation,^30^ transient EBV infection of the other CL components cannot be excluded based on EBER negativity alone. However, EBV persistence exclusively in the nodal cHL component is likely to be an important determinant of cHL fate. Based on these findings, we propose a model for the development of the 4 tumor components from a common ancestral precursor through divergent clonal evolution and the influence of EBV (Figure 3).

**Figure 3.**
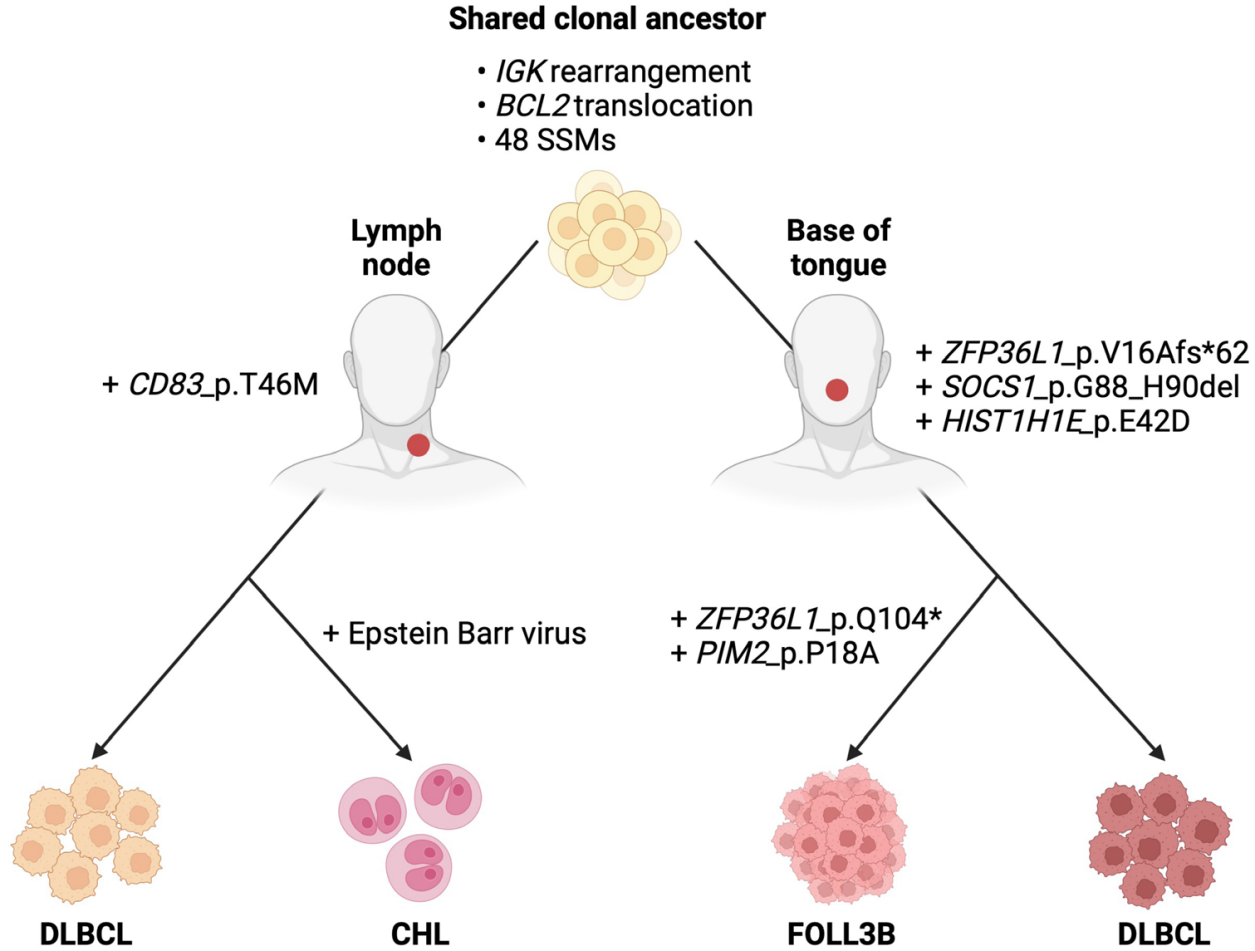
**Proposed model for the development of the 4 tumor components through divergent clonal evolution from a common ancestral precursor.** cHL = classic Hodgkin lymphoma; DLBCL = diffuse large B-cell lymphoma; FOLL3B = follicular lymphoma grade 3B; SSM = simple somatic mutations. Created with Biorender.com.

Due to their heterogeneous nature, the treatment of CL/DL tumors is best individualized. DLBCL manifested in both the cervical lymph node and the tongue, resulting in airway obstruction in this particular case. DLBCL was also suspected to involve the patient’s sizeable cecal mass, supported by subsequent histological confirmation of DLBCL in the colectomy specimen. DLBCL-directed therapy with R-CHOP was therefore deemed most appropriate and yielded a favorable outcome. Further supporting R-CHOP in this patient was its preferred use in FOLL3B, which more closely resembles DLBCL in its clinical behavior, and the adequate activity of the CHOP chemotherapy backbone in cHL.^31^ Maintenance rituximab was omitted because its role in FOLL3B is unclear in contrast to other FL grades.

In conclusion, this is a unique case involving the simultaneous discovery of 2 anatomically separate CLs that led to the diagnosis of 3 distinct GCB-derived lymphoma subtypes of shared clonal origin. Given the paucity of data on optimal therapy for these rare, heterogeneous lymphomas, treatment approach should be individualized, considering all disease components. The increasing recognition of CL/DL underscores recent National Comprehensive Cancer Network recommendations, highlighting an explicit preference for excisional/incisional biopsies on initial diagnosis.^32^ Adequate sampling is necessary to ensure that minor lymphoma components are not missed due to sampling bias. Although Ig-gene rearrangement studies can be used to elucidate the clonal relationship(s) of the different lymphoma entities involved in the clinic, the advantages of next-generation sequencing are emphasized in an era of personalized oncogenomics, where knowledge of mutational landscapes may refine therapeutic strategies of CL/DL in the future.

## ACKNOWLEDGMENTS

The authors are grateful to Dr Hayley Merkeley for inspiring this project and the patient for providing consent for further research analysis of the biopsies. We would also like to thank Dr Brian Skinnider for assistance with image acquisition and Dr Sean Young for technical assistance and advice. We thank Hisae Nakamura for facilitating patient consent for research and Deby Huynh for technical assistance in acquiring the samples for subsequent analyses.

## AUTHOR CONTRIBUTIONS

WA, HAL, and JWC conceptualized the study. MA and JWC acquired and edited the images. SBN, BM, MB, and KM processed samples and performed experiments. WA, JWC, LKH, MA, DWS, and HAL analyzed data. WA and JWC wrote the manuscript. LKH, MA, HAL, DWS, and JWC edited and critically appraised the manuscript. All authors approved the final manuscript.

## DISCLOSURES

The authors have no conflicts of interest to disclose.

## SOURCES OF FUNDING

Next-generation sequencing studies were funded by the BC Cancer Foundation. Dr Alduaij is supported by the University of British Columbia Clinician Investigator Program and the Kuwait Ministry of Health.

## Supplementary Material


