# Supplementary material for: Concurrent Composite Lymphomas Collectively Bearing Three Diagnostic Entities of Shared Clonal Origin
Source: Hemasphere. 2022 Mar 29;6(4):e705. doi: 10.1097/HS9.0000000000000705 (PMC8966960; doi:10.1097/HS9.0000000000000705)
Supplement: Supplementary file 2 [file hs9-6-e705-s002.docx]

**Supplementary Methods- Targeted capture sequencing**

Hybridization capture panels for DLBCL^1^ and Hodgkin/Gray Zone lymphoma^2^ genes were redesigned and synthesized by Twist Bioscience. One-millimetre core fragments were obtained from regions of the formalin-fixed paraffin-embedded (FFPE) tissue blocks corresponding to each separate tumour component as highlighted by the squares in Figures 1a and f. DNA was extracted using the Gentra Puregene Tissue kit (Qiagen) according to manufacturer’s protocol for extraction from FFPE tissue and was treated with S1 nuclease using a previously described protocol^3^. Sequencing libraries were prepared and subjected to hybridization capture using manufacturer protocols and sequenced on the Illumina NovaSeq platform to a mean depth of 1800X. Somatic variants were called using the SLMS-3 consensus variant calling approach (<https://github.com/LCR-BCCRC/lcr-modules>) and annotated with vcf2maf (<https://github.com/mskcc/vcf2maf>). In order to validate the shared clonal or distinct subclonal origin of each somatic variant, all variants identified in any sample were pooled, and the variant allele frequency (VAF) of each variant was calculated for each sample individually. A minimum of three unique reads were required to support the existence of a variant in each sample.

**References:**

1. Rushton CK, Arthur SE, Alcaide M, et al. Genetic and evolutionary patterns of treatment resistance in relapsed B-cell lymphoma. *Blood Adv*. 2020;4(13):2886-2898. doi:10.1182/bloodadvances.2020001696

2. Sarkozy C, Hung SS, Chavez EA, et al. Mutational landscape of gray zone lymphoma. *Blood*. 2021;137(13):1765-1776. doi:10.1182/blood.2020007507

3. Haile S, Corbett RD, Bilobram S, et al. Sources of erroneous sequences and artifact chimeric reads in next generation sequencing of genomic DNA from formalin-fixed paraffin-embedded samples. *Nucleic Acids Res*. 2019;47(2):e12. doi:10.1093/nar/gky1142

**Supplementary Figure 1**

**LN DLBCL**

**LN cHL**

**Tongue**

**DLBCL**

**Tongue FOLL3B**


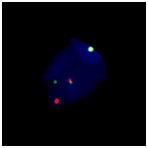

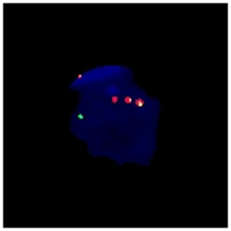

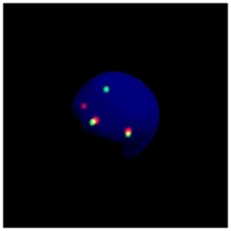

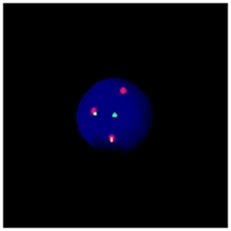


Represenative images of the *BCL2* rerrangement pattern identified by fluorescence *in situ* hybridization using *BCL2* break-apart probes (Metasystems). Nuclei are counterstained with DAPI. Objective magnification: X40. A typical pattern with 2 fused signals, one 3’BCL2 (Red) and one 5’BCL2 (Green) was consistent among all tumor components. LN: lymph node, cHL: classical Hodgkin lymphoma demonstrating a representative Hodgkin Reed-Sternberg cell nucleus, DLBCL: diffuse large B-cell lymphoma, FOLL3B: follicular lymphoma grade 3B.

**Supplementary Table titles**

**Table S1:** Immunohistochemical and cytogenetic characterization of the four tumor components

**Table S2:** List of genes profiled in the customized targeted panel

**Table S3:** Mutations detected in each tumor component

**Table S4:** Mutation annotation format
